# The influence of perceived formative assessment on the learning autonomy of medical students: the chain mediating role of psychological empowerment and positive academic emotions

**DOI:** 10.3389/fpubh.2024.1435432

**Published:** 2024-10-02

**Authors:** Jiali Wang, Guorun Zhou, Juntang Guo, Xiaodong Sun, Lin Sun

**Affiliations:** ^1^School of Psychology, Shandong Second Medical University, Weifang, China; ^2^School of Basic Medical Science, Shandong Second Medical University, Weifang, China; ^3^Department of Endocrinology and Metabolism, Affiliated Hospital of Shandong Second Medical University, Weifang, China

**Keywords:** perceived formative assessment, learning autonomy, psychological empowerment, positive academic emotions, medical students

## Abstract

**Introduction:**

Medical students’ autonomous learning is a cornerstone of their educational journey. Formative assessment is defined as a process to enhance learning and improve academic performance, and the key to the effectiveness of formative assessment is the students’ perceptions of it. The present study investigates the impacts of perceived formative assessment on the learning autonomy of medical students, explores the chain mediating role of psychological empowerment and positive academic emotions, and offers strategies for facilitating medical students’ autonomous learning.

**Methods:**

A cross-sectional investigation was conducted using a convenience sampling method involving 713 medical students (mean age 19.72 ± 1.18 years; 207 male and 506 female students; the proportion of participants is 93.69%) in Shandong Second Medical University. Perceived formative assessment was performed using a self-designed questionnaire of Perceived Formative Evaluation for Medical Students, learning autonomy using the Autonomy in Learning Rating Scale for College Students, positive academic emotions using the General Academic Emotion Questionnaire for College Students, and psychological empowerment of medical students using the Chinese version of Psychological Empowerment Scale (PES). The data were analyzed using descriptive statistics, Pearson’s correlation, multiple regression, and mediation analysis using the SPSS26.0 program and PROCESS3.1.

**Results:**

Perceived formative assessment significantly predicted learning autonomy (*β* = 0.06, *p* < 0.05). It also positively predicted psychological empowerment (*β* = 0.20, *p* < 0.001), and psychological empowerment positively predicted the learning autonomy of medical students (*β* = 0.36, *p* < 0.001). Psychological empowerment also positively predicted positive academic emotions (*β* = 0.64, *p* < 0.001), and positive academic emotions positively predicted learning autonomy (*β* = 0.44, *p* < 0.001). The direct effect value of perceived formative assessment on learning autonomy was 0.18, while the total indirect effect was 0.43. The mediation effect of psychological empowerment on the relationship between perceived formative assessment and learning autonomy was 0.22, and the chain mediation effect of psychological empowerment and positive academic emotions was 0.18, accounting for 30%, 70%, 36%, and 30% of the total effect, respectively.

**Conclusion:**

Perceived formative assessment directly enhances medical students’ learning autonomy. This relationship is partially mediated by psychological empowerment and positive academic emotions. The results suggest that formative evaluation boosts psychological empowerment, which fosters positive academic emotions and further promotes learning autonomy.

## 1 Introduction

Rapid changes in demographics, epidemiology, environmental and behavioral risks, and increasingly complex health issues are creating unprecedented health challenges, which in turn place higher demands on health professionals, necessitating continuous improvement in their professional competence. Nurturing autonomy in learning among medical students is essential for their continuing professional development (1, 2). Autonomy refers to intrinsic motivation and self-governance or self-regulation (3). It is the capacity to take control of one’s own learning process (4) and is also considered a cornerstone of lifelong learning (5). Some research has successfully demonstrated that high learning autonomy plays an important role in increasing medical students’ academic achievement, learning strategies, and study efforts, as well as in reducing students’ exhaustion during the learning process (6, 7). Autonomy also helps build confidence by increasing their clinical decision-making responsibility, which enhances their educational experience (8). Fostering medical students’ autonomous motivation may help deliver healthcare in a humanistic manner by encouraging their use of an autonomy-supportive style of relating to patients (9–11). Learners’ autonomy has primarily been examined within the context of language learning, but research about learning autonomy in medical settings is limited (12). Furthermore, the literature indicates that the factors influencing medical students’ autonomous learning remain relatively unexplored (13). Therefore, investigating the factors influencing medical students’ learning autonomy and offering insights for nurturing their learning autonomy is a meaningful endeavor.

The learning benefits of formative assessment have made it a key consideration in educational reform worldwide (14), and it is also widely used in medical teaching practice and research with the ongoing educational reform in China (15). However, how perceived formative assessment improves learning autonomy in medical students has been less demonstrated. Self-determination theory (SDT) (16, 17) is an organismic-dialectical theory that views human beings as proactive organisms whose intrinsic functioning can be either facilitated or impeded by their social context. This support or hindrance is mediated by the satisfaction of three basic psychological needs: autonomy, competence, and relatedness. According to SDT, autonomy-supportive contexts in which medical students’ basic psychological needs are satisfied will improve their wellbeing and promote intrinsic motivations and self-regulation of extrinsic motivations, leading to more volitional engagement and effectiveness in medical learning. Furthermore, cognitive evaluation theory (CET) (18, 19) hypothesized that social-contextual events (e.g., feedback, communications, and rewards) that promote feelings of competence will not enhance intrinsic motivation unless they are accompanied by an internal perceived locus of causality. In other words, medical students will only experience them as controllers of their learning behavior, leading to intrinsic motivation and learning engagement. Psychological empowerment, as a kind of intrinsic task motivation reflecting a sense of control in relation to one’s work, has been suggested to play a mediating role between supportive management and both employee performance and positive emotions (20). There are still two questions that remain unanswered: Whether psychological empowerment plays a mediating role in the relationship between supportive educational practices and learning autonomy among medical students? How does formative assessment influence learning autonomy through the interaction of psychological empowerment and positive academic emotions? This research, based on self-determination theory (SDT) and cognitive evaluation theory (CET) as a framework, aimed to elucidate the influence of supportive educational practices on medical students’ learning autonomy. It selected formative assessment, the most commonly used method in current medical students’ academic evaluation, as the independent variable, and incorporated psychological empowerment and positive academic emotions into the model to explore the deeper mechanisms underlying the relationship from both cognitive and emotional perspectives. This research will help better understand the mechanisms of formative assessment on the learning autonomy of medical students and provide some implications for fostering medical students’ learning autonomy.

### 1.1 Perceived formative assessment and learning autonomy

One study has shown that formative assessment plays a crucial role in promoting learners’ autonomy (21). Formative assessment is defined as encompassing all those activities undertaken by teachers, and/or by their students, which provide information to be used as feedback to modify the teaching and learning activities in which they are engaged (22, 23). Since formative assessment is highly student-focused and interactive, provides quality feedback and feed-forward, and is adaptive, it allows students to autonomously learn problem-solving procedures and enables them to take an active role in their own learning by challenging them (17, 24, 25). Therefore, it has been recognized as a beneficial strategy for enhancing their self-regulation and empowering them to become autonomous learners (23, 26–30). A growing body of research provides extensive evidence that, if well perceived by students, formative assessment has the potential to contribute to improving students’ learning. According to Harks et al. (31), formative assessment affects students’ engagement in learning through students’ perception of its usefulness. Kyaruzi et al. (32) found that students’ perceptions of the quality of teacher feedback delivery and perceived scaffolding positively predicted students’ feedback use, whereas perceived monitoring negatively predicted feedback use. Based on this, we propose the following hypothesis:

*Hypothesis I*: Perceived formative assessment can positively predict learning autonomy in medical students.

### 1.2 Perceived formative assessment, psychological empowerment, and learning autonomy

Psychological empowerment is defined as intrinsic task motivation reflecting a sense of control in relation to one’s work and cognitive orientation to one’s work role (33), and it is shaped by the contextual environment and contributes to enhancing feelings of self-efficacy and autonomy (34–37). Extant theoretical and empirical studies suggested that psychological empowerment may be an important mediator between organizational environment and work performance, commitment, and engagement (20, 38–43). According to CET, people must experience their behavior as self-determined for intrinsic motivation to be evident. This requires inner resources that are typically the result of prior developmental supports for autonomy and competence (44). Relevant research confirmed the mediating effect of psychological empowerment in the relationship between perceived formative assessment and learning. Studies showed that formative assessment improves students’ feeling of empowerment to take control of their own learning processes (45–47) and intrinsic motivation (48). Previous research has also revealed that psychological empowerment is related to autonomy. For example, Watkins’ study found a significant relationship between psychological empowerment and professional autonomy (49). Myrick suggested that psychological empowerment fosters autonomy, choice, control, and responsibility, which, in turn, creates empowered schools (50). Psychological empowerment will be more influential in beliefs about one’s own skills and efforts when it involves exercising control, participating in decision-making, or engaging in problem-solving (51).

Based on the above, we propose the following hypothesis:

*Hypothesis II*: Psychological empowerment plays a mediating role in the relationship between perceived formative assessment and learning autonomy.

### 1.3 Perceived formative assessment, positive academic emotions, and learning autonomy

An extensive body of research has revealed that positive academic emotions are considered a potential mediating variable of the relationship between formative assessment and learning autonomy. Pekrun’s (52) cognitive and motivational model postulates that positive emotions have a positive influence on learners’ self-regulation. The relationship between positive academic emotions and learning autonomy has been well-established in research. Some studies have shown how positive emotions are related to a range of autonomy-related variables such as competency beliefs (53), mastery and performance approach goals (54, 55), study effort, learning strategies, self-regulation (56), and engagement (57, 58). Studies show that perceived teacher’s supportive feedback was positively related to individual levels of academic enjoyment (53) and emotional resilience (59). Recently, a longitudinal study showed that perceived learning control has positive effects on academic enjoyment (60). Thus, we propose the following hypothesis:

*Hypothesis III*: Positive academic emotions play a mediating role in the relationship between perceived formative assessment and learning autonomy.

**Figure 1:**
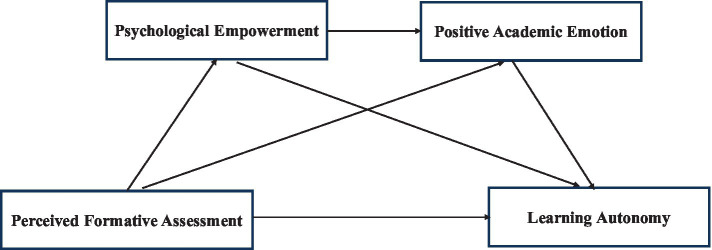
Chain mediating model hypothesis of psychological empowerment and positive academic emotions in the relationship between perceived formative assessment and learning autonomy of medical students.

The chain mediating model hypothesis model is shown in Figure 1.

### 1.4 The chain mediating role of psychological empowerment and positive academic emotions on perceived formative assessment and learning autonomy

Psychological empowerment may mediate the relationship between perceived formative assessment and positive academic emotions of medical students. A meta-analysis of psychological empowerment has revealed that perceived support in an organization is one of the antecedents of psychological empowerment and that psychological empowerment acts as a motivational factor that may generate emotional reactions (20). Furthermore, positive academic emotions may mediate the relationship between psychological empowerment and learning autonomy of medical students. A longitudinal study showed that positive affect mediates the relationship between psychological empowerment and employee outcomes (61). According to SDT, when an individual is given an opportunity for self-direction, basic psychological needs are fulfilled and then intrinsic motivation is enhanced, thus leading to self-integration (44).

Drawing from the above framework and findings of related studies, we propose the following hypothesis:

*Hypothesis IV*: Psychological empowerment and positive academic emotions together play a chain mediating role in the relationship between perceived formative assessment and learning autonomy.

The theoretical model used in this study is shown in Figure 1.

## 2 Materials and methods

### 2.1 Participants

We estimated the sample size using a prior analysis using G*Power 3.1, selecting the multiple linear regression method (62) and setting the effect size at a medium level (*f*^2^ = 0.15), the statistical power (1-*β*) = 0.95, and type I errors (*α*) = 0.001. Based on these parameters, the estimated sample size is not less than 208. A cross-sectional survey via the online survey website Wenjuanxing was conducted using the convenience sampling method to investigate medical students at Shandong Second Medical University located in Shandong Province. A total of 761 questionnaires were collected. After excluding invalid questionnaires (i.e., failed to complete all answers, options are inconsistent, and item responses were quicker than the reasonable rate of 2 s per item), 713 questionnaires were considered valid (93.69*%* valid response rate) (63). All subjects were between the ages of 18 and 23 years of age (M = 19.72; SD = 1.18). The sample included 207 men (29.03%) and 506 women (70.97%). Of these, 351 were first-year students (49.23%), 189 were second-year students (26.51%), and 173 were third-year students (24.26%). Participants were enrolled in majors related to clinical medicine (437, 61.29%), medical technology (185, 25.95%), and other fields (91, 12.76%).

### 2.2 Measures

#### 2.2.1 Perceived formative evaluation questionnaire for medical students

This study measured medical students’ perceived formative assessment using a self-designed questionnaire of Perception of Formative Evaluation for Medical Students. The questionnaire was constructed following the standardized procedure of questionnaire development. The questionnaire consists of 41 items, which are divided into two subscales: perception of assessment and perception of feedback. Each item was rated using a Likert scale ranging from “1” (never perceived) to “4” (often perceived). The higher the score, the higher the level of perceived formative assessment. Confirmatory factor analysis showed that the CMIN/DF value of the questionnaire was 3.067, the CFI value was 0.880, and the RMSEA value was 0.083, indicating acceptable structural validity. Cronbach’s *α* of the questionnaire was 0.95.

#### 2.2.2 Autonomy in learning rating scale for college students

The Autonomy in Learning Rating Scale for College Students used in this study was developed by Yuan (64). This scale consists of 61 items and includes four dimensions: self-regulation, learning strategies, content and environment, and learning motivation. The scale uses a 5-point Likert scoring method (from 1 = “Not like me at all” to 5= “very much like me”); a higher score indicates a higher level of learning autonomy. Cronbach’s *α* of the scale was 0.98 for this study.

#### 2.2.3 General academic emotion questionnaire for college students

The General Academic Emotion Questionnaire for College Students was developed by Ma (65). The questionnaire consists of 88 items and four dimensions: negative activating emotions, positive activating emotions, negative deactivating emotions, and positive deactivating emotions. It uses a 5-point Likert scale rating method (from 1 = “strongly disagree” to 5 = “strongly agree”). A higher dimension score indicates a higher level of experienced academic emotion. This study used the positive emotions items (38 items) to evaluate the academic positive emotions of medical students. Cronbach’s α of the scale was 0.95 for this study.

#### 2.2.4 Psychological empowerment scale

The Psychological Empowerment Scale (PES) was originally constructed by Spreitzer (33), and the Chinese version was revised by Li-Chaoping et al. (66). To better adapt the scale to the learning context, some expressions were modified. For instance, “work” was changed to “learning” and “department” was changed to “class.” The scale consists of four dimensions including meaning, self-efficacy, self-determination, and impact, with a total of 12 items. It adopts a 5-point Likert scoring method (from 1 = “strongly disagree” to 5 = “strongly agree”). A higher score indicates a higher level of psychological empowerment. The scale has been shown to have good reliability and validity. Cronbach’s α of the scale was 0.93 for this study.

### 2.3 Date analysis

Data analysis was performed using the SPSS26.0 program and PROCESS3.1. Harman’s single-factor analysis was first conducted to check for potential common method bias. The reliability of each of the scales used in the current study was evaluated using Cronbach’s α coefficient. Pearson’s correlation analysis was conducted to explore the association among variables. Subsequently, PROCESS 3.1 (Model 6) was used to test the chain mediation effect of PE and PAE. The bias-corrected percentile bootstrap method was used to estimate the 95% confidence interval with 5,000 repeated sampling (67). The effects are considered to be statistically significant if the CI does not contain zero.

## 3 Results

### 3.1 Multicollinearity and common method variance

An exploratory factor analysis was used to test for possible common method bias by incorporating all questionnaire items. The results showed 21 factors with eigenvalues greater than 1. The first factor accounted for 30.16%, which is less than 40% (68), indicating no serious common method deviation in the data of this study.

### 3.2 Correlation between variables

A significant correlation was found between formative assessment, psychological empowerment, positive academic emotions, and learning autonomy of medical students (Table 1), and the results supported conducting a further mediating effects analysis (69).

**Table 1:** Correlation analysis of study variables (*N* = 713).

	*Mean*	*SD*	PFA	PE	PAE	LA
PFA	126.90	12.60	—			
PE	43.91	7.66	0.20^**^	—		
PAE	134.03	19.93	0.12^**^	0.64^**^	—	
LA	204.49	39.36	0.18^**^	0.65^**^	0.68^**^	—

PFA, perceived formative assessment; PE, psychological empowerment; PAE, positive academic emotions; LA, learning autonomy. *p < 0.05, **p < 0.01.

### 3.3 The mediating effects of psychological empowerment and positive academic emotions

PROCESS 3.1 was used to analyze the mediating role of psychological empowerment and positive academic emotions between perceived formative assessment and learning autonomy of medical students, controlling the variables such as major and hometown. The results of the regression analysis (Table 2) showed that perceived formative assessment had a significant positive predictive effect on learning autonomy (*β* = 0.06, *p* < 0.05), confirming hypothesis I; perceived formative assessment positively predicted psychological empowerment (*β* = 0.20, *p* < 0.001), and psychological empowerment positively predicted the learning autonomy of medical students (*β* = 0.36, *p* < 0.001), confirming hypothesis II; and psychological empowerment positively predicted positive academic emotions (*β* = 0.64, *p* < 0.001), and positive academic emotions positively predicted learning autonomy (*β* = 0.44, *p* < 0.001), confirming hypothesis IV. The effect of perceived formative assessment on positive academic emotions was not significant when the chain mediating effect was tested (*β* = 0.02, *p* > 0.05).

**Table 2:** Regression model of the effect of PFA on LA in medical students (*N* = 713).

Variables	*β*	*t*	*P*	*LLCI*	*ULCI*	*R^2^*	*F*
PFA → PE	0.20	5.47	0.00^***^	0.08	0.17	0.04	10.84^***^
PFA → PAE	0.02	0.70	0.49	−0.57	0.12	0.42	126.30^***^
PE → PAE	0.64	21.78	0.00^***^	1.51	1.81		
PAE → LA	0.44	13.23	0.00^***^	0.74	1.00	0.55	172.69^***^
PE → LA	0.36	10.70	0.00^***^	1.50	2.17		
PFA → LA	0.06	2.23	0.03^*^	0.02	0.34		

PFA, perceived formative assessment; PE, psychological empowerment; PAE, positive academic emotions; LA, learning autonomy. *p < 0.05, ***p < 0.001.

**Figure 2:**
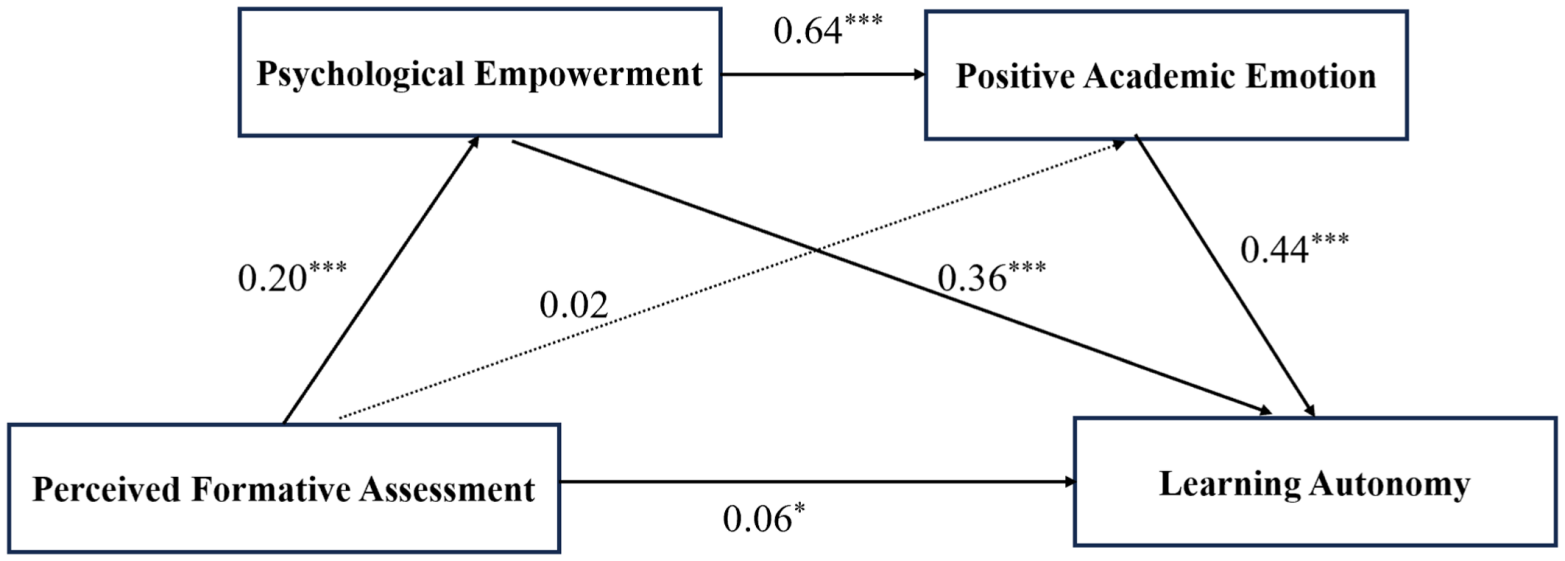
Illustrated pathway showing the chain mediating effect.

The bootstrap method was used to test the chain mediating effect of psychological empowerment and positive academic emotions between perceived formative assessment and learning autonomy in medical students. Table 3 shows the 95% confidence intervals of the bootstrap sampling test for each path, and none of the bootstrapped 95% confidence intervals for these indirect effects contained a value of 0, indicating that the indirect effects are significant. Specifically, the total indirect effect was significant, with an indirect effect value of 0.43; the mediating effect of psychological empowerment on perceived formative assessment and learning autonomy in medical students was significant, with a mediating effect value of 0.22; and the chain mediating effect of psychological empowerment and positive academic emotions in the relationship between perceived formative assessment and learning autonomy in medical students was also significant, with a mediating effect value of 0.18, accounting for 70, 36, and 30% of the total effect, respectively. The detailed pathway model is shown in Figure 2.

**Table 3:** The mediating effects of PE and PAE (*N* = 713).

		Effect	Boot SE	Boot LLCI	Boot ULCI	Ratio to total effect
Direct effect		0.18	0.08	0.02	0.34	0.30
Indirect effect	Total indirect effect	0.43	0.09	0.27	0.60	0.70
	PFA PE LA	0.22	0.05	0.14	0.32	0.36
	PFA PE PAE LA	0.18	0.04	0.11	0.26	0.30
Total effect		0.61	0.11	0.38	0.83	

PFA, perceived formative assessment; PE, psychological empowerment; PAE, positive academic emotions; LA, learning autonomy.

## 4 Discussion

This study explored the chain mediating effects of psychological empowerment and positive academic emotions in the relationship between perceived formative assessment and learning autonomy of medical students by constructing a chain mediation model. The results indicate a significant positive correlation between perceived formative assessment, psychological empowerment, positive academic emotions, and learning autonomy of medical students. Further regression analysis results also indicate a significant positive predictive effect of perceived formative assessment on the learning autonomy of medical students, confirming our first hypothesis. When psychological empowerment and positive academic emotions entered into the equation, the direct effect of perceived formative assessment on the learning autonomy of medical students changed. The findings of the mediation test revealed that psychological empowerment and positive academic emotions partially mediated the effect of perceived formative assessment on the learning autonomy of medical students. The literature revealed that limited studies have investigated the specific pathways between perceived formative assessment and learning autonomy in medical students. This study provided empirical evidence supporting the impact of formative assessment on learning autonomy and extended these findings to the education of medical students. The results help to a better understanding of the mechanisms between formative assessment and learning autonomy in medical students from both cognitive and emotional perspectives. Since this study is a cross-sectional study, it cannot confirm causality.

### 4.1 The relationship between perceived formative assessment and learning autonomy in medical student

The results of this study showed that perceived formative assessment significantly predicted the learning autonomy of medical students, which is consistent with the findings of previous studies (21, 70). Although supporters of formative assessment agree that it improves students’ learning autonomy, research has found that students and teachers have different perceptions of formative assessment (70, 71), and teachers’ autonomous teaching predicted students’ autonomous learning through students’ perceptions of it (72). Therefore, students’ perception and utilization of formative assessment provided by teachers play an important role in enhancing learning autonomy. More perceived use of formative assessment is associated with more feelings of autonomy (73). According to th self-determination theory (SDT), students have basic psychological needs for autonomy, competence, and relatedness. When students perceive that their behavior emanates from the self and is self-authored, they experience it as volitional, and it fulfills their basic psychological needs, thus enabling them to develop autonomous motivation (44, 74). Formative assessment is very student-centered and supportive, fulfilling students’ basic psychological needs when perceived well by students (24, 25). This perception and fulfillment allow students to exert control over their learning, make their own decisions, and then enable students to become autonomous learners.

### 4.2 The chain mediating role of psychological empowerment and positive academic emotions in the relationship between perceived formative assessment and learning autonomy in medical students

The results of this study showed that perceived formative assessment impacts the learning autonomy of medical students through the chain of psychological empowerment and positive academic emotions. This is the main contribution of this research. The results showed that perceived formative assessment indirectly affects the learning autonomy of medical students through two pathways: psychological empowerment and the chain mediating effect of psychological empowerment and positive academic emotions.

First, the results of this study found that perceived formative assessment positively predicts psychological empowerment, and psychological empowerment positively predicts the learning autonomy of medical students. This path accounts for 36% of the total effects, indicating that psychological empowerment is an important mediating factor in the relationship between perceived formative assessment and learning autonomy in medical students. Research into psychological empowerment has reported strong evidence confirming its role as a mediating and motivational factor in organizational and community psychology (20, 75). Based on previous research, Llorente-Alonso et al. (20) posited that high-performance managerial practices, oriented toward offering workers greater access to support, resources, and learning, act as empowering elements. In addition, individuals’ perception of real rewards and support enables workers to feel empowered. Formative assessment is precisely a practice that “encourages students and gives them a greater sense of ownership” by deeply involving them in strategies such as personal goal-planning, monitoring, and reflection, thus giving learners “the power to oversee and steer their own learning” (76). Cauley and McMillan (48) also discovered that formative assessment encourages students and gives them a greater sense of ownership in instructional activities. Psychological empowerment positively predicted the learning autonomy of medical students. This result is consistent with previous studies (49, 50), suggesting that psychological empowerment plays an important role in motivating and activating students to regulate and be responsible for their own learning. Psychological empowerment can be viewed as a cognitive and motivational process by which students develop learning autonomy based on perceived formative assessment (20, 33, 37).

Second, the result of this study showed that positive academic emotions mediated the relationship between psychological empowerment and learning autonomy of medical students. This path (perceived formative assessment→psychological empowerment→positive academic emotions→learning autonomy) accounts for 30% of the total effect, indicating that perceived formative assessment influences learning autonomy through the chain of psychological empowerment and positive academic emotions. On the one hand, psychological empowerment positively predicted positive academic emotions, indicating that students who experience higher levels of psychological empowerment also experience more positive academic emotions. Previous studies found that learning engagement of university students, which is led by psychological empowerment (77), mediates the relationship between perceived assessment and learning satisfaction (78).

On the other hand, the result of this study showed that positive academic emotions positively predict the learning autonomy of medical students. This result is consistent with previous findings (56, 57). Many studies have confirmed that achievement emotions affect the cognitive, motivational, and regulatory processes mediating learning and achievement (52, 56). Positive academic emotions can help focus attention, strengthen intrinsic and extrinsic motivation, facilitate students’ self-regulation of learning (56), and enhance students’ learning autonomy. This result indicates that educators need to value positive academic emotions in teaching, especially for medical students who face a heavier burden of schoolwork coupled with high standards of performance, making them more susceptible to experiencing negative emotions (79, 80), even burnout (81).

Interestingly, the results of this study found that perceived formative assessment does not predict positive academic emotions directly. This is inconsistent with previous studies (53). This result can be explained by Pekrun’s control-value theory of achievement emotions, which postulates that the affective impact of social environments is mediated by control and value appraisals (82). This insignificant result may also be due to our exclusive focus on positive emotions. Future research could include both positive and negative emotions together to illustrate this question. This result illustrated that the influence of perceived formative assessment on positive academic emotions is completely mediated by psychological empowerment.

## 5 Contributions and limitations

These findings revealed the relationship among perceived formative assessment, psychological empowerment, positive academic emotions, and learning autonomy in medical students. The findings of this study provide insight into the psychological mechanisms underlying the influence of perceived formative assessment on learning autonomy. They extend the findings into medical education and enrich the existing literature on formative assessment and learning autonomy. This finding indicates that educators and teachers should consider the design of formative assessment from the perspectives of both teachers and students, pay attention to promoting students’ psychological empowerment, and value positive academic emotions. These findings provide empirical evidence about the relationship among perceived formative assessment, psychological empowerment, positive academic emotions, and learning autonomy in medical students, which may provide some useful insights for further exploration of the relationship between these four variables.

Despite its contributions to the existing literature, this study also has several limitations. First, this study used a cross-sectional questionnaire method, which makes it impossible to clarify the causal relationship between variables. A longitudinal research design should be applied to further validate the findings of this research in the future. Second, the mediation variables in this research were only psychological empowerment and positive academic emotions and did not include other possible factors. Third, this research only studied the factors from the perspective of individuals, without considering environmental factors and the individual-environmental interactions.

## 6 Conclusion

The finding of this study demonstrated the relationship between perceived formative assessment and learning autonomy in medical students and the chain mediated effect of psychological empowerment and positive academic emotions. Specifically, perceived formative assessment has a direct positive impact on the learning autonomy of medical students and an indirect positive impact through the chain mediating factors of psychological empowerment and positive academic emotions.

## Data Availability

The raw data supporting the conclusions of this article will be made available by the authors, without undue reservation.
